# Measuring configural spatial knowledge: Individual differences in correlations between pointing and shortcutting

**DOI:** 10.3758/s13423-023-02266-6

**Published:** 2023-03-17

**Authors:** Chuanxiuyue He, Alexander P. Boone, Mary Hegarty

**Affiliations:** University of California, Santa Barbara, CA USA

**Keywords:** Direction estimation, Wayfinding, Individual differences, Psychometrics

## Abstract

People use environmental knowledge to maintain a sense of direction in daily life. This knowledge is typically measured by having people point to unseen locations (judgments of relative direction) or navigate efficiently in the environment (shortcutting). Some people can estimate directions precisely, while others point randomly. Similarly, some people take shortcuts not experienced during learning, while others mainly follow learned paths. Notably, few studies have directly tested the correlation between pointing and shortcutting performance. We compared pointing and shortcutting in two experiments, one using desktop virtual reality (VR) (N = 57) and one using immersive VR (N = 48). Participants learned a new environment by following a fixed route and were then asked to point to unseen locations and navigate to targets by the shortest path. Participants’ performance was clustered into two groups using K-means clustering. One (lower ability) group pointed randomly and showed low internal consistency across trials in pointing, but were able to find efficient routes, and their pointing and efficiency scores were not correlated. The others (higher ability) pointed precisely, navigated by efficient routes, and their pointing and efficiency scores were correlated. These results suggest that with the same egocentric learning experience, the correlation between pointing and shortcutting depends on participants’ learning ability, and internal consistency and discriminating power of the measures. Inconsistency and limited discriminating power can lead to low correlations and mask factors driving human variation. Psychometric properties, largely under-reported in spatial cognition, can advance our understanding of individual differences and cognitive processes for complex spatial tasks.

**Supplementary Information:**

The online version contains supplementary material available at 10.3758/s13423-023-02266-6.

## Introduction

Learning the layout of a new environment, that is spatial knowledge acquisition, is a fundamental cognitive function. Humans rely on spatial knowledge to maintain a sense of direction while locomoting through different environments and planning routes to goal locations. Environmental spatial knowledge encompasses different kinds of knowledge, including landmark, route, and configural knowledge (McNamara, 2013; Siegel & White, 1975). Configural knowledge is assumed to integrate all spatial information into a globally consistent mental representation. Compared to landmark and route knowledge, acquiring configural knowledge shows the largest individual differences (Ishikawa & Montello, 2006; Peer et al., 2021; Weisberg & Newcombe, 2018). It is critical to investigate these individual differences using valid and reliable measures (Newcombe et al., 2023) to advance our understanding of configural knowledge.

Configural knowledge acquisition is typically measured by direction estimation or shortcutting tasks after giving participants a controlled experience of learning routes through a new environment from an egocentric perspective. In direction estimation tasks, participants are asked to point to unseen target locations from different locations and perspectives in the newly learned environment (judgments of relative direction). The fidelity of configural knowledge is measured by average absolute pointing error, that is, the angular disparity between the correct direction and the participant’s estimate, averaged across trials (e.g., Ishikawa & Montello, 2006; Meilinger et al., 2014; Schinazi et al., 2013). In shortcutting tasks, participants are asked to take the shortest path to goal locations in the environment, and the measure of performance is wayfinding efficiency, or directness of the path, measured by comparing the path taken to the optimal (shortest) traversable path to the goal location, again averaging over trials (e.g., Gagnon et al., 2016, 2018; Gallistel, 1990; Hartley et al., 2003; He et al., 2019; Tolman, 1948). Note that knowledge of the route that people learn during the learning phase is not sufficient to perform either of these tasks, so they measure how well participants have inferred configural knowledge from the egocentric learning experience. Moreover, in some research paradigms, the walls disappear during wayfinding, so “shortcutting” means straight-line navigation (e.g., Chrastil & Warren, 2013; Foo et al., 2005; Warren et al., 2017). In others, participants cannot go through the walls, and shortcutting means route-based shortcutting (e.g., Chrastil & Warren, 2015; Hartley et al., 2003; He et al., 2021). In the present study, we use the term *shortcutting* to refer to route-based shortcutting.

To examine individual differences in acquiring configural knowledge, researchers have typically used either shortcutting efficiency (e.g., Gallistel, 1990; Hartley et al., 2003) or angular error^1^ (e.g., Hegarty et al., 2006; Ishikawa & Montello, 2006; Meilinger et al., 2014; Weisberg et al., 2014; Weisberg & Newcombe, 2018), or have measured pointing and shortcutting performance based on different environments (e.g., Malanchini et al., 2020). Even when both pointing and shortcutting were measured after learning the same environment (e.g., He et al., 2019, 2021; Labate et al., 2014), researchers under-reported the relationship between these measures. It is assumed that they are equally valid and perhaps interchangeable measures of configural knowledge. However, the cognitive demands of estimating the direction to a goal location and of taking the shortest path to that location may not be equivalent. In a route-based shortcutting paradigm, path choices are constrained by the street or path network of an environment (Pagkratidou et al., 2020). In some instances, the shortest path to a goal location may involve temporarily turning away from the direction to the target. Moreover, the ability to point accurately to a goal location is not necessary for efficient wayfinding. For example, participants can take advantage of wormholes to take shortcuts without realizing the physical impossibility of the environment (Muryy & Glennerster, 2018; Warren et al., 2017).

Examining the differential cognitive demands and individual differences in two tasks can thus inform debates on the nature of configural knowledge. One view is that configural knowledge is metrically accurate and globally consistent (Carpenter et al., 2015; Gallistel, 1990; O’Keefe & Nadel, 1978; Siegel & White, 1975; Tolman, 1948), like a physical or cartographic map. Another view is that configural knowledge is labeled graph knowledge, in which close locations are connected with coarse, local metric information (direction and distance) but not metrically consistent across the whole environment (Chrastil & Warren, 2015; Foo et al., 2005; Warren, 2019). Other views are that this distinction is subject to individual differences (Weisberg & Newcombe, 2018) or that map-based knowledge and graph-based knowledge coexist, with the use of different types of knowledge depending on environmental characteristics and navigational demands (Peer et al., 2021). Chrastil and Warren (2015) have proposed that the route-based shortcutting task measures graph-based knowledge and the pointing task measures map-based knowledge.

Here, we examine the correlations between pointing and shortcutting after the same learning experience to address the question of whether they are interchangeable measures of configural knowledge. To address this question, the first step is to examine the psychometric properties of the two measures, as this may affect the correlation between the measures. Based on classical test theory (Novick, 1966; Wilson, 2005), previous researchers have assumed equal difficulty and adequate discriminating power across the items in these measures. The equal difficulty or internal consistency assumption is that participants’ performance on one trial can predict their performance on the other trials. Note that internal consistency is one type of measurement reliability. The adequate discriminating power assumption is that the test items can effectively distinguish people with a high trait level from people with a low trait level. However, the difficulty across trials and discriminating power may vary due to differential availability and saliency of navigation cues such as landmarks and street structure in different trials (Caduff & Timpf, 2008; Röser et al., 2012; Sorrows & Hirtle, 1999), and people may be differentially susceptible to these factors (Andersen et al., 2012; Barhorst-Cates et al., 2021; Coutrot et al., 2022; He et al., 2021; Lawton, 2001; Weisberg & Newcombe, 2016). Ignoring reliability may mislead researchers to conclude a dissociation between the abilities measured by two tasks based on a low correlation, when, in fact, that low correlation is due to the low reliability of the individual measures (Ackerman & Hambrick, 2020; Hedge et al., 2018; Parsons et al., 2019; Newcombe et al., 2023). Ignoring inadequate discriminating power leads to the pitfall that the reported results are only applicable to a subset of the population, whereas others are out of scope due to ceiling or floor effects (Cramer & Howitt, 2005; Kang & MacDonald, 2010; Newcombe et al., 2023).

A secondary goal of the present study was to study the generalizability of our findings across navigation scenarios with and without body-based senses. Previous research has highlighted the importance of body-based internal sensory cues (i.e., proprioception, vestibular system, and motor efference) in acquiring map-based configural knowledge. For example, Anastasiou and colleagues (2022) suggested that without body-based cues, people may just acquire graph-based knowledge, whereas, with these cues, and corresponding path integration processes, people gain more precise knowledge including metric distance and direction.

In the present study, we examined the internal consistency and discriminating power of pointing and shortcutting measures after people learned the layout of environments, how these psychometric properties influence correlations between the measures, and the interpretation of these correlations. We also examined psychometric properties and correlations separately for more and less able spatial learners. We conducted two experiments, one in a desktop virtual environment, in which people used a mouse and keyboard to navigate, and one in an ambulatory immersive virtual environment.

## The present studies

### Method

#### Participants

##### Desktop virtual reality study 

Seventy-two undergraduate students (38 female) participated in this study for course credit. Eight female participants were unable to complete the task due to motion sickness, two were excluded because they failed to reach the target on more than 30% of trials, and five male participants were excluded due to technical issues. Fifty-seven (28 female, median age 19 years, range 18–25 years) were included in the final analysis.

##### Immersive virtual reality study 

Fifty-one undergraduate students (27 female) participated in this study for course credit. Three female participants were unable to complete the task due to technical issues or misinterpreting the instructions. Forty-eight participants (24 female, median age 19 years, range 18–25 years) were included in the final analysis.

A statistical power analysis showed that with N = 48, we could detect a correlation of .4 (a medium effect size: Cohen, 1988) with alpha = .05 and power = 0.80.

#### Materials

##### Desktop virtual reality study

- **Virtual maze**

**Fig. 1:**
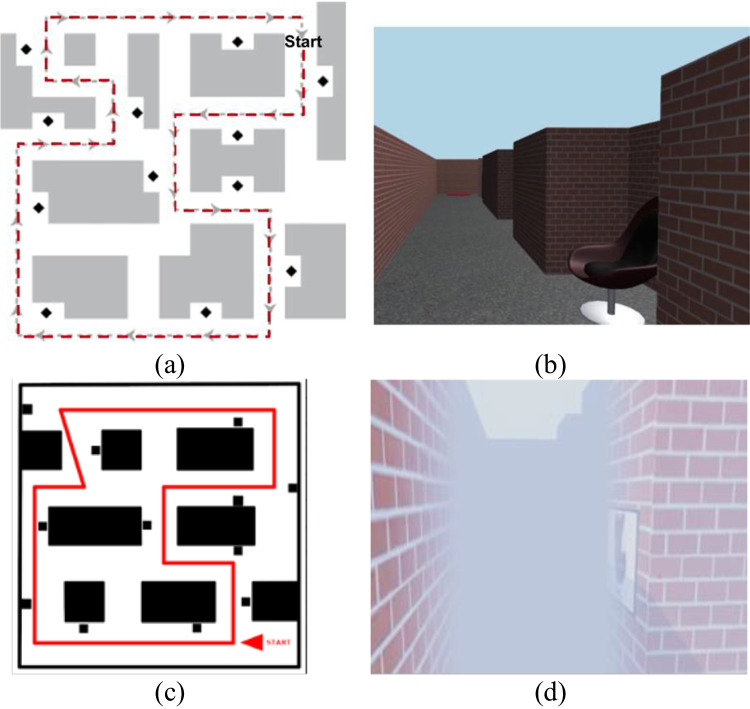
**a** Map of the virtual environment used by Boone et al. (2019), the red dashed line indicates the route people use to learn the environment during the learning phase. **b** Participants’ view of the desktop environment. **c** Map of the immersive virtual environment, the red line indicates the route for learning. **d** Participants’ view of the immersive virtual environment

The 11 $\times$ 11 m experimental maze, as shown in Fig. 1a and b, was taken from Boone et al. (2019) (Maze 1). Twelve landmarks were placed in alcoves in the maze (see Fig. 1a). During the learning phase, people learned the environment by taking a fixed tour of the maze five times.

The experiment was administered using a Dell XPS with a GeForce GTX 1070 graphics card. The environment was presented using Unity3D and displayed on a 24-in. LCD monitor (289.9 × 531.4 mm display area), with a refresh rate of 60 Hz at a resolution of 1,920 × 1,080 and a viewing distance of approximately 1 m.

- **Direction estimation task**

**Fig. 2:**
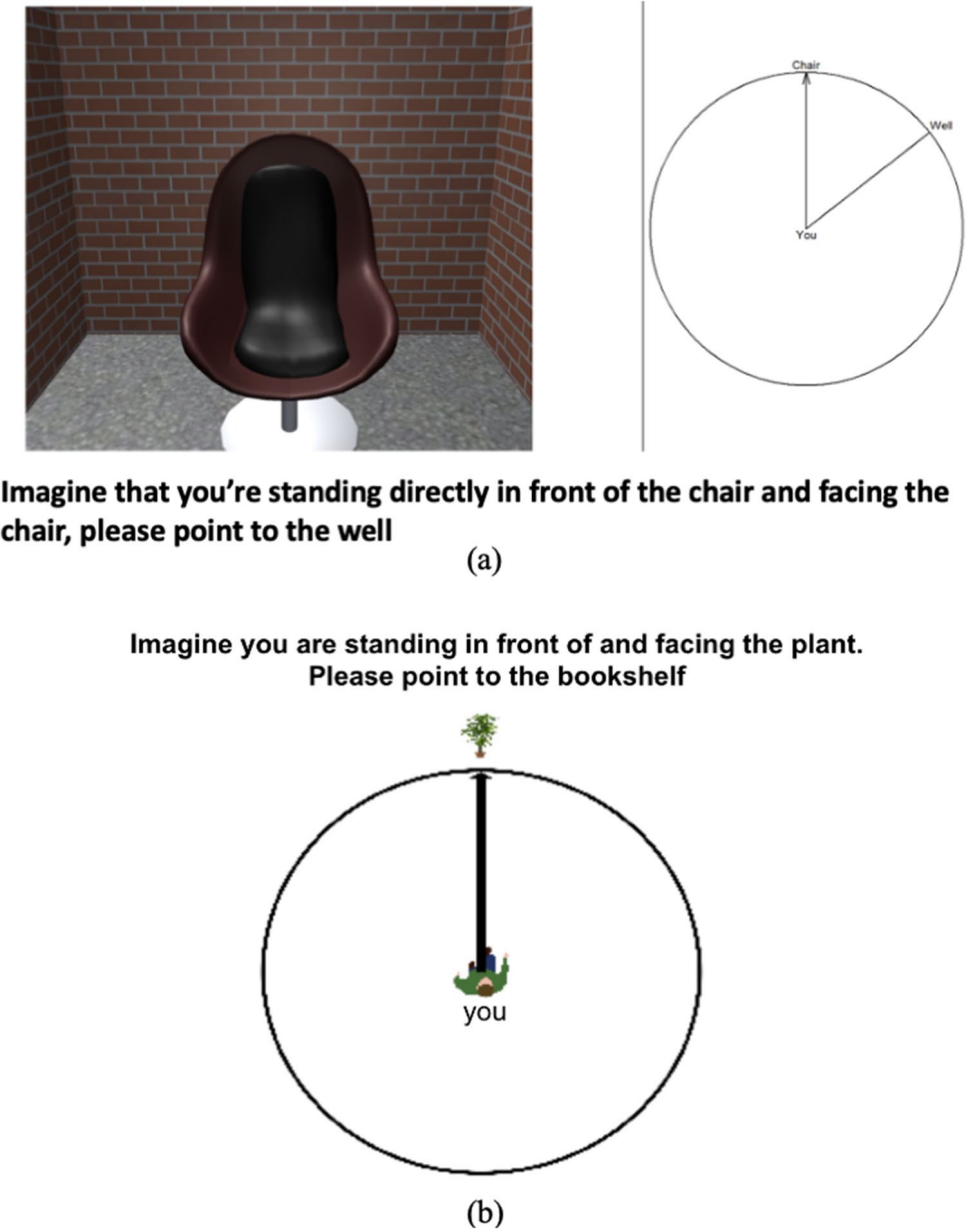
**a** The sample item on the instruction screen for the direction estimation task in the desktop study. **b** The sample item for the direction estimation task in the immersive study

The direction estimation task was conducted using E-prime 2.0 (Schneider et al., 2012) and was administered twice for each participant, once before the shortcutting task (Pointing Phase I) and once after the shortcutting task (Pointing Phase II). On each trial, participants were shown an image of a landmark (starting landmark) on the left half of the screen. An arrow circle was displayed on the right half of the screen (see Fig. 2a). Participants were instructed to imagine being in the maze and facing the starting landmark and to indicate the direction to another (target) landmark (which was not visible from the current location). For example, in one trial, participants were shown a picture of the chair and were asked to point to the well (see Fig. 2a). They indicated the target landmark by dragging a line (a rotating “pointer”) on the displayed arrow circle. There were 27 trials, and the score on this task was the average angular error across trials (Pointing Error). Twenty of these trials used the same starting and target landmarks as the shortcutting task.^2^

- **Shortcutting task**

In the shortcutting task, participants were positioned at different locations in the maze and instructed to navigate to target landmarks using the shortest path. There were 20 shortcutting trials, which were presented in random order. The shortest path on each trial was at least 25% and on average 51% shorter than the learned route. Participants had 40 seconds to complete each trial. At the end of each trial (finding the target or timing out), participants were transported to the starting location of the next trial.

##### Immersive virtual reality study

- **Virtual maze**

The 7 $\times$ 6.5 m experimental maze, as shown in Fig. 1c–d, had a similar structure to the desktop study and the same 12 landmarks. However, given the physical space constraint of the laboratory, it has a smaller scale and we replaced the 3D objects with pictures of these objects on the walls. Condensing the structure leads to higher visibility, compared to the desktop environment, which means participants can gain more visual information about the structure of the environment at some locations. To make this environment more comparable to the desktop study, we added fog (see Fig. 1d). The fog obscured vision beyond 2.5 m and the clarity decreased linearly between 1 and 2.5 m.

The immersive virtual environment was displayed using an HTC VIVE Pro Eye VR head-mounted display (HMD) with a Dual OLED 3.5-in. diagonal display (1,440 $\times$ 1,600 pixels per eye or 2,880 $\times$ 1,600 pixels combined), a 90-Hz refresh rate, and a 110° field of view capable of delivering high-resolution audio through removeable headphones. In addition to the HMD, the VR interface included two HTC VIVE wireless handheld controllers for interacting with the experiment and four HTC Base Station 2.0 infrared tracking sensors for large-scale open space tracking. The system was equipped with wireless room tracking via a 60-GHz WiGig VIVE Wireless adapter and was run on an iBuyPower desktop computer powered by an eight-core, 3.60 GHz Intel core i9-9900K central processing unit (CPU), an NVIDIA GeForce RTX 2070 Super graphics processing unit (GPU) with 16 GB of system memory. Participants physically walked in the environment while wearing the HMD.

- **Direction estimation task**

As shown in Fig. 2b, the direction estimation task in the immersive VR study was similar to the desktop study and was run on the desktop, except that the task was programmed in Unity and had 24 trials in total. The 24 trials had the same landmark combinations as the shortcutting task but switched the starting and target landmarks. For example, in the shortcutting task, participants were asked to start from the bookshelf to navigate to the plant, but in the direction estimation task, participants were asked to face the plant and point to the bookshelf. We implemented this change to reduce the impact of the direction estimation task on the shortcutting task. On each trial (as shown in Fig. 2b), participants were instructed to imagine being in the maze and facing the starting landmark, and to indicate the direction to another (target) landmark. They indicated the target landmark by dragging a line (a rotating “pointer”) on the displayed arrow circle (see Fig. 2b). The score on this task was the average angular error across trials (Pointing Error).

- **Shortcutting task**

The shortcutting task was similar to the desktop study except that participants physically walked in the environment and had 24 trials. Participants had 30 seconds for each trial. Between trials, to disorient participants from the previous trial and relocate participants to a new starting location, they were placed in an empty space with floor and visual checkpoints. They were asked to walk to a random checkpoint and then to another checkpoint, placing them in the position and orientation to start a new trial. The 24 trials were selected to ensure the following criteria: (1) each landmark was the start location twice; (2) each landmark was the target at least once but no more than three times, and (3) the shortest path on each trial was at least 30% and on average 49% shorter than the learned route.

#### Procedure

The local Institutional Review Board (IRB) reviewed and approved both studies as adhering to ethical guidelines. In the desktop study, all participants completed the experiment in a lab cubicle alone, with an experimenter giving instructions. In the immersive study, all participants completed the experiment in the immersive VR lab alone, with one experimenter giving instructions and one experimenter handling the computers. For both studies, after giving informed consent, participants were trained to use the digital arrow circle on the computer screen to indicate directions. Their comprehension of how to indicate directions was checked by having them use the arrow circle to point to two visible objects in the experiment room.

**Fig. 3:**
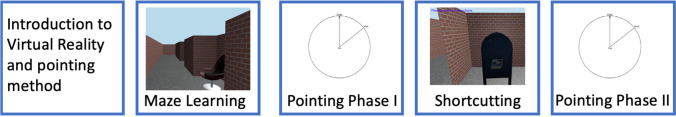
The order of tasks in the two experiments

Participants then practiced using the active navigation controls (Desktop: keyboard and mouse; Immersive: headset and controllers) in a training maze.^3^ Next, participants were placed in the experiment environment maze with red arrows and followed these arrows to learn a route through the virtual environment five times, saying the name of each object aloud as it came into view the first time. After participants followed this route five times, three spatial tasks were administered in a fixed order: (1) direction estimation task – Phase I, (2) shortcutting task, and (3) direction estimation task – Phase II, see Fig. 3.^4^ Finally, participants were debriefed.

All analyses were carried out using Python scripts.

### Results

#### Overall performance

Descriptive statistics, including the internal consistency of the measures, are presented in Table 1. Participants were generally successful in reaching the target within the time limit in both the desktop and immersive VR studies, except for one trial in the desktop study in which 17 of the 57 participants (30%) were unsuccessful; this trial was excluded from wayfinding analyses. Participants were successful on 92.9% of the remaining trials in the desktop study and on 94.5% of the trials in the immersive study. Travel Efficiency was defined as the ratio of the distance traveled to the distance of the shortest traversable path on each trial. If a participant took the shortest path on every trial, their efficiency would be 1, and if they took the learned path on every trial, their efficiency would be 2.54 on average for the desktop VR maze (i.e., the average learned route efficiency) and 2.19 for the immersive VR maze. Travel efficiency for the unsuccessful trials was replaced by the average learned route efficiency.^5^

**Table 1:** Descriptive statistics for pointing error and efficiency for all participants

	Study	Mean	SD	Min	Max	Skewness	Kurtosis	# of Trials	Reliability
Pointing Error (Phase I)	Desktop	73.71	23.22	18.85	105.26	-0.69	-0.27	27	0.83
	Immersive	64.58	27.45	8.21	102.97	-0.67	-0.69	24	0.87
Efficiency	Desktop	1.81	0.39	1.00	2.51	-0.32	-0.93	19	0.72
	Immersive	1.56	0.39	1.04	2.58	0.61	-0.48	24	0.87
Pointing Error (Phase II)	Desktop	65.37	23.40	15.89	104.67	-0.25	-0.83	27	0.84
	Immersive	52.90	28.11	12.49	99.97	0.07	-1.46	24	0.90

As shown in Table 1, the average pointing error (angular error) in Phase I direction estimation was 74.71° (*SD* = 23.22) and 64.58° (*SD* = 27.45), respectively, for the desktop and immersive environments. Although relatively poor, average performance across all participants was significantly better than chance (90°), one-sample *t*(56) = -5.30, *p* < 0.001, *d* = -.70, 95% CI = [67.54, 79.87] in Desktop and one-sample *t*(47) = -6.42, *p* < 0.001, *d* = -.93, 95% CI = [56.61, 72.55] in Immersive.

**Fig. 4:**
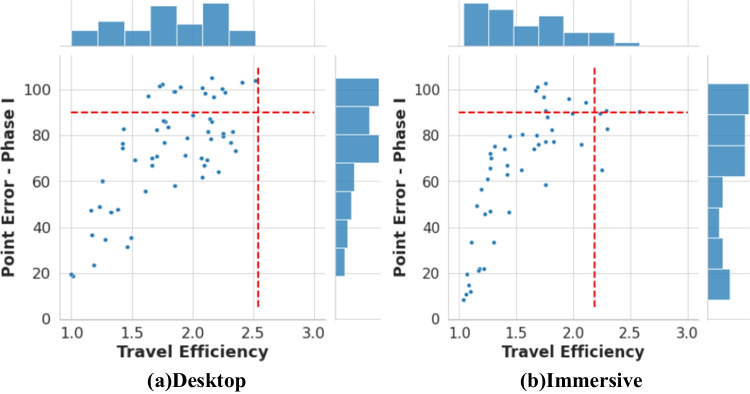
Scatter plots for the Pointing Error (Phase I) in the direction estimation task Phase I and Travel Efficiency in the shortcutting task in the desktop study (**a**) and in the immersive study (**b**). The red horizontal line indicates the chance level performance: 90°. The red vertical line indicates the average efficiency of taking learned routes on every trial: 2.54 (**a:** in the desktop study) and 2.19 (**b:** in the immersive study)

The average travel efficiency score across trials was 1.81 for the desktop VR environment and 1.56 for the immersive VR environment. Therefore travel distance was, on average, significantly shorter than the learned route (Desktop: one-sample *t* test (56) = -14.02, *p* < 0.001, *d* = -1.86, 95% CI = [1.71, 1.91]; Immersive: one-sample *t* test (47) = -10.99, *p* < 0.001, *d* = -1.59, 95% CI = [1.45, 1.68]). Notably, in the shortcutting trials, most participants took paths that were shorter than the learned route, although their pointing performance was relatively poor. This is illustrated in Fig. 4 in which the horizontal line indicates chance-pointing performance and the vertical red line indicates the efficiency score of a person who always takes the learned route.

The observed and disattenuated correlations between the measures are shown in Table 2. Disattenuated correlations take the internal consistency (i.e., permutation-based split-half estimation)^6^ of the measures into account using Formula (1) (Parsons et al., 2019; Spearman, 1904) where ${r}_{observed}$ is the observed correlation between two measures, ${r}_{xx}$ and ${r}_{yy}$ are internal consistency scores of two measures and ${r}_{disattenuated}$ is calculated as follows:

1 $${r}_{disattenuated}=\frac{{r}_{observed}}{\sqrt{{r}_{xx}\times {r}_{yy}}}$$

**Table 2:** The observed and disattenuated correlation table for all participants

Disattenuated	Travel Efficiency	Pointing Error - Phase I	Pointing Error - Phase II
Observed			
Desktop			
Travel Efficiency	-	.92***	.97***
Pointing Error - Phase I	.71***	-	.93***
Pointing Error - Phase II	.76***	.78***	-
Immersive			
Travel Efficiency	-	0.86***	0.90***
Pointing Error - Phase I	0.74***	-	0.88***
Pointing Error - Phase II	0.79***	0.78***	-

Values below the diagonal, in the bottom left are the observed correlations and values above the diagonal in the top right are the disattenuated correlations corrected using Eq. (1). *** indicates *p* <.001

Participants who were more accurate at pointing at both phases were also more efficient in shortcutting trials, and this relationship is particularly strong in the case of the disattenuated correlations, which correct for internal consistency. However, these results mask individual differences between participants, which are presented in the next section.

#### Individual differences: Low-spatial participants versus high-spatial participants

A K-means clustering analysis was conducted on three measures (efficiency, Phase I, and Phase II pointing errors) to categorize participants as having low or high-spatial ability.^7^ Note that two was the optimal number of clusters based on the elbow and the silhouette method (see Online Supplemental Materials (OSM) for additional information). Descriptive statistics and internal consistency for each measure are shown in Table 3, separately for these two groups.^8^

**Table 3:** Descriptive statistics and internal consistency for measures

	Spatial Ability	Mean	SD	Min	Max	Skewness	Kurtosis	# of Trials	Internal Consistency
Desktop									
Pointing Error (Phase I)	High	50.36	19.62	18.85	83.04	-0.05	-1.11	27	0.80
	Low	86.32	13.01	61.74	105.26	-0.13	-1.23		0.40
Efficiency	High	1.38	0.23	1.00	1.85	0.20	-0.68	19	0.65
	Low	2.04	0.24	1.52	2.51	-0.24	-0.75		0.01
Pointing Error (Phase II)	High	40.50	14.41	15.89	69.41	0.22	-0.87	27	0.71
	Low	78.82	14.60	52.56	104.67	0.10	-1.10		0.51
Immersive									
Pointing Error (Phase I)	High	44.02	23.09	8.21	75.34	-0.17	-1.46	24	0.85
	Low	85.14	11.16	58.77	102.97	-0.42	-0.24		0.12
Efficiency	High	1.24	0.14	1.04	1.55	0.42	-0.52	24	0.57
	Low	1.89	0.28	1.44	2.58	0.76	-0.22		0.57
Pointing Error (Phase II)	High	28.27	12.04	12.49	51.07	0.41	-1.09	24	0.62
	Low	77.54	14.25	45.35	99.97	-0.70	-0.09		0.47

For the desktop study, only including the corresponding trials 19 trials in the pointing tasks do not change the conclusions. See OSM

For low-spatial participants, in the desktop study (*N* = 37), the average pointing error before the shortcutting task (Phase I pointing) (86.32°, *SD* = 13.01°), was not significantly different from chance (90°), one-sample *t* (36) = -1.72, *p* = 0.09, *d* = -0.28, 95% CI = [81.98, 90.66]. Moreover, these participants’ pointing performance across trials was not reliable (internal consistency = 0.40). However, their average travel efficiency score was 2.04, which was significantly shorter than the learned route (Efficiency = 2.54), one-sample *t*(36) = -12.77, *p* < 0.001, *d* = -2.1, 95% CI = [1.96, 2.12], suggesting some ability to take novel paths that were more efficient than the learned route, even though they pointed at chance and their pointing performance was not consistent across trials. Similarly, in the immersive study (*N* = 24), low-spatial participants’ pointing performance (85.14°, SD = 11.16°) was better than chance, one-sample *t* (23) = -2.13, *p* = 0.04, d = -0.44, 95% CI = [80.42, 89.85], but close to chance. Their pointing performance was also not reliable (internal consistency = 0.12). However, their average travel efficiency (1.89) was significantly more efficient than the learned route (2.19), one-sample *t* (23) = -5.25, *p* < .001, d = -1.07, 95% CI = [1.77, 2.01], suggesting some ability to find shorter paths than the learned route, even though their pointing performance was close to chance and was not consistent across trials.

**Fig. 5:**
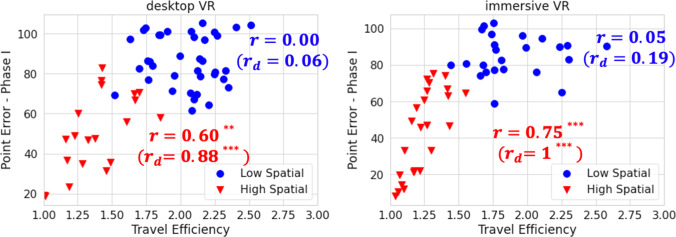
Scatter plots, observed correlation(*r*), and disattenuated correlation(${r}_{d}$) between pointing and shortcutting for high- and low-spatial groups in the desktop study and the immersive study. *Note:* For the high-spatial participants, indicated by the red (triangular) points, the correlations between pointing and shortcutting are almost 1. *** indicates *p* <.001. However, for low-spatial participants, indicated by the blue (circular) points, the correlations were not significantly different from zero. The low correlations were partially driven by the low internal consistency of the measures. A full correlation table is in the OSM TS1

As shown in Fig. 5, for low-spatial participants, the observed correlations between Pointing Error (Phase I) and shortcutting are not significant (Desktop: *r(35)* = 0.00, *t*(35) = 0.02, *p* = .98, 95% CI = [-.32, .33]; Immersive: *r(22)* = .05, *t*(22) = 0.23, *p* = .82, 95% CI = [-.36, .44]). These correlations were partially driven by the low internal consistency of both measures, suggesting that individual-level correlation coefficients were attenuated by measurement variance unrelated to true between-individual variances. After correcting for the internal inconsistency of the measure, the disattenuated correlations between the Pointing Error (Phase I) and shortcutting were still not significant (see Fig. 5); that is, low-spatial participants’ pointing performance cannot predict their shortcutting performance.

For high-spatial participants (Desktop: *N* = 20; Immersive: *N* = 24), pointing performance in the first phase was highly correlated with shortcutting. (Desktop: *r(14)* = .60, *t*(14) = 3.03, *p* = .01, 95% CI = [.15, .85]; Immersive: *r(22)* = .75, *t*(22) = 5.35, *p* < .001, 95% CI = [.50, .89]) with higher correlations after correcting for the internal inconsistency (see Fig. 5). The disattenuated correlations for the high and low-spatial groups were significantly different (Desktop: *Fisher’s z* = 4.43, *p* < .001, *Zou*’s 95% CI = [-1.28,-0.38]; Immersive: *z* = 7.95, *p* < .001, *Zou’s* 95% CI = [-1.22,-0.44]).

Note that in the immersive study, the internal consistency for shortcutting was 0.57, which is relatively low. The relatively low internal consistency, in this case, was driven by the close-to-ceiling performance. That is, the variance for each trial was determined by a small number of participants who did not get the perfect efficiency score (efficiency of 1) and so there was limited variance to correlate between trials.

## General discussion

We examined the relation between pointing and shortcutting performance after the same egocentric learning experience in two studies, one using desktop VR and the other using immersive VR. The results of these studies are consistent. In both studies, the correlation between shortcutting and pointing depends on participants’ learning ability, as well as the internal consistency and discriminating power of the measures. The high-spatial groups across studies were generally good at both shortcutting and pointing and the correlation between shortcutting and pointing was high for these groups; the low-spatial groups had poor pointing performance but took novel and efficient routes, and shortcutting and pointing were not significantly correlated for these groups.

Relations between shortcutting and pointing were affected by both the discriminability and internal consistency of the measures. In terms of discriminability, we observed a tension between the difficulty of the pointing task for the low-spatial group and the difficulty of the shortcutting task for the high-spatial group (see Fig. 5). The desktop environment was relatively difficult to learn, given the amount and type of learning experience given in these studies, such that we observed a floor effect for the low-spatial group in the pointing task. The immersive environment was easier to learn, but resulted in a close-to-ceiling effect for the high-spatial group in the shortcutting task. Given the wide range of individual differences in large-scale spatial cognition, we recommend that future researchers examine the discriminating power of their measures and use measures that can distinguish across the full range of ability. They may need to combine multiple measures to assess all levels of environmental learning ability.

Low-spatial participants showed low internal consistency in their pointing and shortcutting performance, while high-spatial participants showed relatively low internal consistency in their shortcutting performance in immersive VR, which attenuated the observed correlation between the two measures (Ackerman & Hambrick, 2020; Hedge al., 2018; Parsons et al., 2019). The item-level variance may be driven by (1) inconsistent accuracy of mental representations for different locations in the environment (e.g., landmarks near the boundary or aligned with specific orientations may be easier to learn), (2) differential availability of navigational cues in different trials, and (3) participants’ differential sensitivity to these cues (e.g., Andersen et al., 2012; Barhorst-Cates et al., 2021; Coutrot et al., 2022; He et al., 2021, Newcombe et al., 2023). Investigating the effect of these factors calls for future studies. Our study highlights that these underlying cognitive processes are masked if researchers do not investigate their instruments by first examining measurement reliability.

These analyses help us advance our understanding of the nature of configural knowledge, specifically on whether this is best characterized as labeled graph knowledge or metrically accurate survey knowledge (Foo et al., 2005; Gallistel, 1990; Kuipers et al., 2003; O’Keefe & Nadel, 1978; Peer et al., 2021; Warren, 2019). Our results show that pointing performance is accurate and is correlated with shortcutting for high-spatial participants, but pointing performance is less accurate and not correlated with shortcutting for low-spatial participants. This suggests that the high-spatial group may have acquired both types of knowledge, whereas the low-spatial group only acquired graph knowledge with this amount of learning experience.

Our pointing task provided only one view of the environment in each trial and did not allow people to look around before estimating the direction. Low-spatial participants' relatively poor performance in pointing might also reflect difficulty orienting themselves in the environment based on this limited information. Future research, using a more immersive pointing measure will help distinguish whether poor pointing performance by this group is due to a poor cognitive map of the environment or an inability to locate themselves in this cognitive map. The present study provides one way of examining the measures, and the key point is that underlying knowledge measured for different people may change if the paradigms and trials are changed.

To conclude, instead of assuming that pointing and shortcutting are interchangeable measures of environmental knowledge, our studies show that it is critical to examine psychometric properties, including reliability and discriminability, before selecting measures or interpreting the correlations. Psychometric properties are largely under-reported in the spatial cognition domain but can advance our understanding of individual differences and should be an important foundation of research on cognitive processes underlying complex spatial tasks.

### Supplementary Information

Below is the link to the electronic supplementary material.

Supplementary file1 (DOCX 385 KB)

## Data Availability

The datasets generated during and/or analysed during the current study are available in the ConfiguralSpatialKnowledgeMeasurement repository [https://github.com/CarolHeChuanxiuyue/ConfiguralSpatialKnowledgeMeasurement.git].

## Footnotes

1 Map reconstruction and straight line distance estimation are also common individual differences measures in environmental learning studies (Ishikawa & Montello, 2006; Ruginski et al., 2019; Schinazi et al., 2013; Weisberg et al., 2014), but these two measures are beyond the scope of this paper.

2 For the analysis only including the corresponding subsets, see the Online Supplementary Materials (OSM). The conclusions do not change.

3 The training mazes had different structure to the mazes used in the tasks and had no landmarks. In the desktop study, participants practiced using mouse and keyboard to follow arrows along the floor until comfortable. In the immersive study, participants practiced walking to three gray bubbles and using the controllers to click bubbles. They were also given the time to freely explore the training maze until comfortable.

4 We also included an onsite direction estimation task (pointing in the environment) for the desktop study after pointing Phase II for exploratory analyses, which are not included in this paper.

5 Note that using this substituting method, participants get a penalty (or their efficiency was deflated) if they fail to locate a target, because taking the learned route is an inefficient method in the current paradigm. Another method is removing both the unsuccessful shortcutting trials in calculating efficiency and the corresponding trials in the pointing task. The conclusions do not change if we use this alternative method. Detailed results based on the alternative method are shared online (https://github.com/CarolHeChuanxiuyue/ConfiguralSpatialKnowledgeMeasurement.git).

6 Data are repeatedly randomly split into two halves 5,000 times. The final internal consistency is the average of the 5000 split-half reliability estimates. (Parsons et al., 2019).

7 An alternative way to group participants is using a median split on their pointing performance in the first phase, however, the main conclusions of the paper do not change if we use this method.

8 The low-spatial groups were unsuccessful in finding the target on more trials (2.04 trials in the desktop; 2.08 trials in the immersive version) than the high-spatial groups (0.10 trials in the desktop; 0.54 trials in the immersive version). The efficiency score captures these differences as described in the method section, so we only examined the efficiency scores in the following analysis.
