# Supplementary material for: Measuring configural spatial knowledge: Individual differences in correlations between pointing and shortcutting
Source: Psychon Bull Rev. 2023 Mar 17;30(5):1802–13. doi: 10.3758/s13423-023-02266-6 (PMC10716069; doi:10.3758/s13423-023-02266-6)
Supplement: Supplementary file 1 — Supplementary file1 (DOCX 385 KB) [file 13423_2023_2266_MOESM1_ESM.docx]

**Supplemental Material**

**Section 1: K-means Clustering and Full Correlation Table for All Measures**

K-means clustering was used to cluster participants into low and high spatial groups based on their scores on direction estimation Phase I, Phase II and shortcutting task (efficiency and Phase I and II pointing error). K-means clustering aims to cluster observations into k clusters so that the total intra-cluster variation is minimized, whereas observations from different clusters are as dissimilar as possible. The intra-cluster variance is measured by the sum of squared Euclidean distances. It is one of the simplest and most computationally efficient partitioning methods (Forgy, 1965; Lloyd, 1982). The k-means clustering was conducted using the Python sklearn package and all variables were normalized to the range between 0 and 1 to give equal importance to all features. The optimal number of clusters, which was two, was determined by the elbow method and Silhouette score (See Figure S1). The cluster results were plotted in Figure 5 (in the main text) and Figure S2, where Cluster 1 had poor performance in all three measures, whereas Cluster 2 had good performance in all three measures. Thus, Cluster 1 was labelled as low spatial group, and Cluster 2 was lablelled as high spatial group.

We included both pointing phases because we noticed individual differences in improvement in the pointing task after the shortcutting task (See Figure S3). To what extent participants can continue to develop configural knowledge during the shortcutting task is another indicator of environmental learning ability because the shortcutting task provided a chance to freely explore the environment and make active decisions (Chrastil & Warren, 2013; Munion, et al., 2019; König, et al., 2019; Gagnon, et al., 2018).

The correlations among three measures for high and low spatial groups in two studies are shown in Table S1.


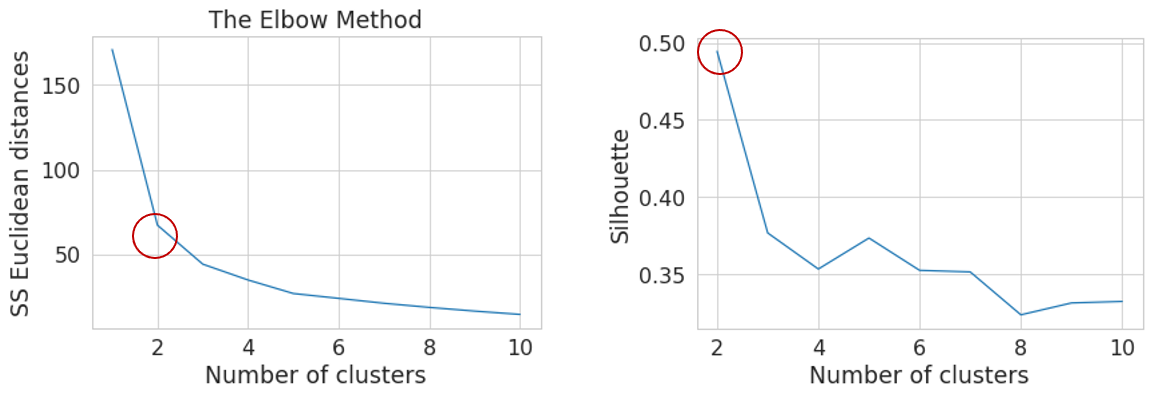


(a) Desktop Virtual Environment


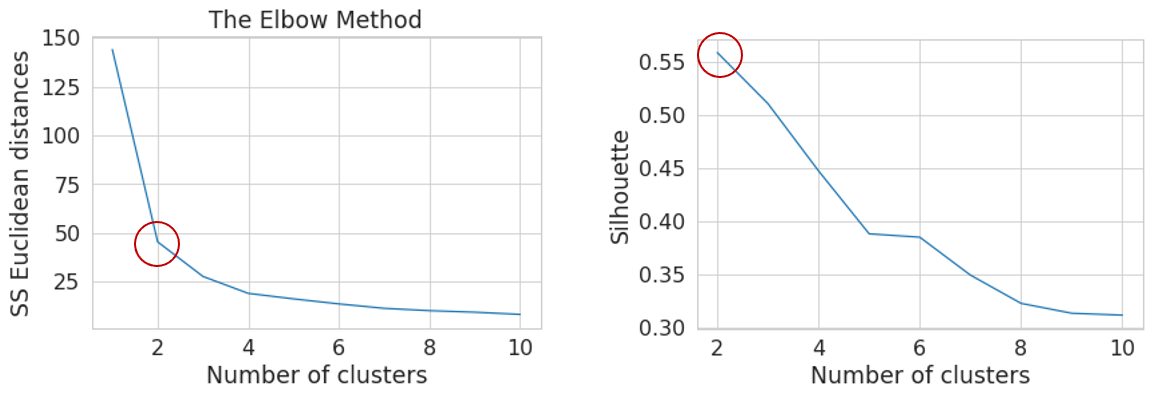


(b) Immersive Virtual Environment

Figure S1: Elbow and Silhouette Plots for (a) the Desktop Study and (b) the Immersive Study. The elbow, where the sum of the squared distances falls suddenly, indicates the optimal K, which were 2 in both studies. A higher Silhouette score indicates the data point is more compact within the cluster and more far away from the other clusters. Thus, the maximum Silhouette value indicates the optimal K, which were 2 in both studies.


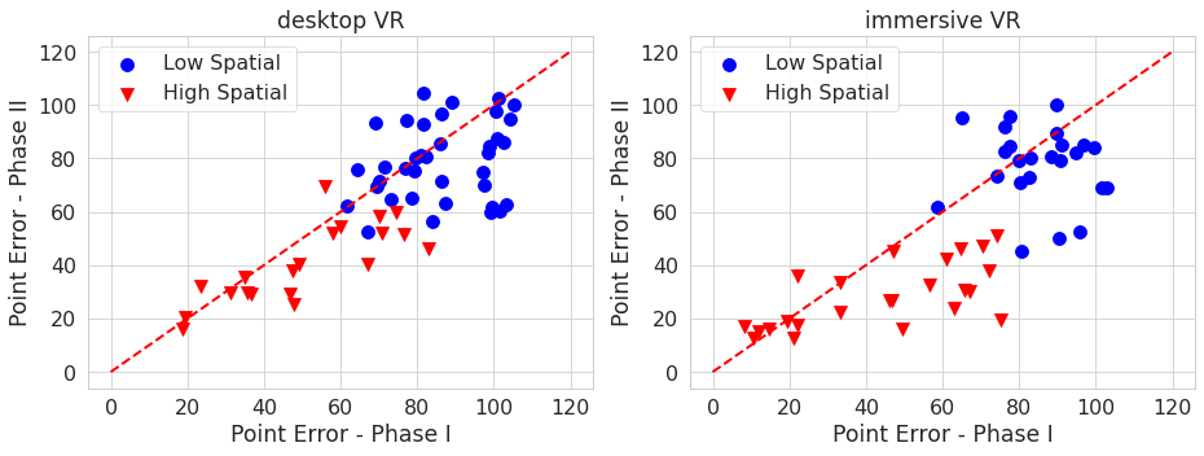


(a) (b)

Figure S2: Scatter plots for the Pointing Error Phase I and Phase II in (a) the Desktop Study and (b) the Immersive Study, Labeled for High and Low Spatial Groups. When points fall on the red lines, the corresponding participants have the exactly same average errors for Phase I and Phase II. Most people’s data points were lower than the line, indicating that they improved on the pointing task from phase 1 to phase 2.

*Table S1*

*Observed and Disattenuated Correlation Tables for High and Low Spatial Participants, including Phase I and Phase II pointing performance.*

| 1. **Desktop VR study (non-matched trials)^3^** | | | | | | |
| --- | --- | --- | --- | --- | --- | --- |
|  | **High-Spatial (N=27)** | | | **Low-Spatial (N=30)** | | |
| Disattenuated  Observed | **Travel Efficiency** | **Pointing Error - Phase I** | **Pointing Error - Phase II** | **Travel Efficiency** | **Pointing Error - Phase I** | **Pointing Error - Phase II** |
| **Travel Efficiency** | - | 0.88*** | 0.94*** | - | 0.06 | 1*** |
| **Pointing Error - Phase I** | 0.64** | - | 1*** | 0.00 | - | 0.52** |
| **Pointing Error - Phase II** | 0.64** | 0.78*** | - | 0.18 | 0.23 | - |

| **(b)immersive VR study (matched trials)** | | | | | | |
| --- | --- | --- | --- | --- | --- | --- |
|  | **High-Spatial (N=24)** | | | **Low-Spatial (N=24)** | | |
| Disattenuated  Observed | **Travel Efficiency** | **Pointing Error - Phase I** | **Pointing Error - Phase II** | **Travel Efficiency** | **Pointing Error - Phase I** | **Pointing Error - Phase II** |
| **Travel Efficiency** | - | 1*** | 0.93*** | - | 0.19 | 0.16 |
| **Pointing Error - Phase I** | 0.75*** | - | 0.91*** | 0.05 | - | -0.41* |
| **Pointing Error - Phase II** | 0.55** | 0.66*** | - | 0,08 | -0.10 | - |

| **(c)Desktop VR study (matched trials)** | | | | | | |
| --- | --- | --- | --- | --- | --- | --- |
|  | **High-Spatial (N=14)** | | | **Low-Spatial (N=43)** | | |
| Disattenuated  Observed | **Travel Efficiency** | **Pointing Error - Phase I** | **Pointing Error - Phase II** | **Travel Efficiency** | **Pointing Error - Phase I** | **Pointing Error - Phase II** |
| **Travel Efficiency** | - | 0.93*** | 0.58* | - | 0.22 | 0.70*** |
| **Pointing Error - Phase I** | 0.61* | - | 1*** | 0.07 | - | 0.55*** |
| **Pointing Error - Phase II** | 0.30 | 0.63* | - | 0.24 | 0.28 | - |

Notes:

1. For all tables, the bottom left are the observed correlations and the top right half are the disattenuated correlations corrected using Equation (1).

2. *: *p* < .05; **: *p* < .01; ***: *p* < .001

3. Desktop non-matched table (a) is based on all 27 pointing and 19 shortcutting trials. Desktop matched table (c) is based on the 19 pointing trials matched to the shortcutting trials.

**Section 2: Subset of Direction Estimation Tasks in Desktop VR study**

***Subsets of the Direction Estimation Task Phase I & Phase II***

In the desktop VR experiment, there were more pointing trials than shortcutting trials. The following analyses for the direction estimation tasks (Phase I & Phase II) only consider the subset of trials in the direction estimation tasks that match the 19 trials analyzed in the shortcutting task. These results are consistent with the results reported in the main text.

***Descriptive Statistics for Overall Performance***

As shown in Table S2, the average pointing error (angular error) in the Phase I direction estimation was 77.37 (SD = 25.05) deg. Although relatively poor, average performance across all participants was significantly better than chance (90 deg), one-sample *t*(56) = -3.81, *p*<0.001, *d*= -.50, 95% CI = [70.72, 84.01]. Consistent with the conclusions reported in the main text, in the shortcutting trials, most participants took paths that were shorter than the learned route although their pointing performance was relatively poor. Moreover, participants who were more accurate at pointing at both phases were also more efficient in shortcutting trials, and this relationship is particularly strong in the case of the disattenuated correlations, which are corrected for internal consistency. However, these results mask individual differences between participants, which are presented in the next section.

*Table S2:*

*Descriptive statistics for pointing error and efficiency for all participants based on the 19 pointing trials that corresponded to the wayfinding trials.*

|  | Mean | SD | Min | Max | Skewness | Kurtosis | # of Trials | internal consistency |
| --- | --- | --- | --- | --- | --- | --- | --- | --- |
| Pointing Error (Phase I) | 77.37 | 25.05 | 16.16 | 116.58 | -0.73 | -0.2 | 19 | 0.79 |
| Efficiency | 1.81 | 0.39 | 1.00 | 2.51 | -0.32 | -0.93 | 19 | 0.72 |
| Pointing Error  (Phase II) | 66.41 | 24.98 | 14.32 | 113.11 | -0.14 | -0.70 | 19 | 0.80 |

*Table S3.*

*The Observed and Disattenuated Correlation Table for All participants. Pointing measures are based on the 19 trials that correspond to the wayfinding trials.*

| Disattenuated  Observed | **Travel Efficiency** | **Pointing Error - Phase I** | **Pointing Error - Phase II** |
| --- | --- | --- | --- |
| **Travel Efficiency** | - | .88*** | .92*** |
| **Pointing Error - Phase I** | .66*** | - | .90*** |
| **Pointing Error - Phase II** | .70*** | .71*** | - |

*Note:* Values below the diagonal, in the bottom left, are the observed correlations and values above the diagonal in the top right are the disattenuated correlations corrected using Equation (1).

A K-means clustering was conducted again based on updated the pointing errors for only the matched trials (see the elbow and silhouette plots in Figure S3 and the clustering results in Figure S4). Descriptive statistics and internal consistency for each measure are shown in Table S4, separately for the high and low spatial groups.


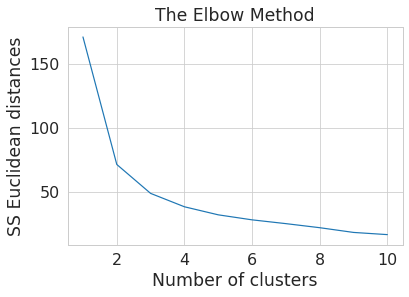

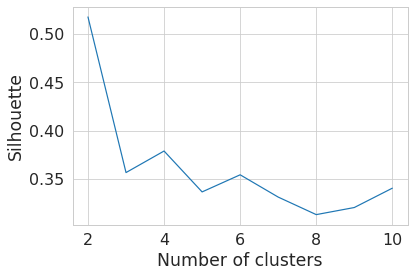


Figure S3: Elbow and Silhouette Plots for the Desktop Study Based on Matched Trials


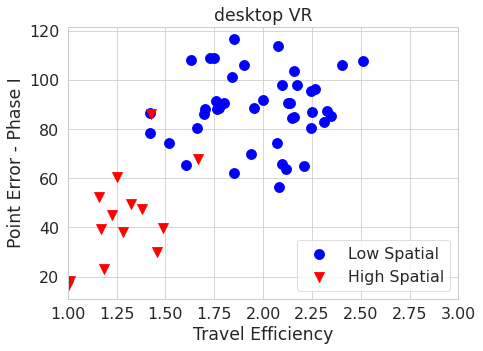

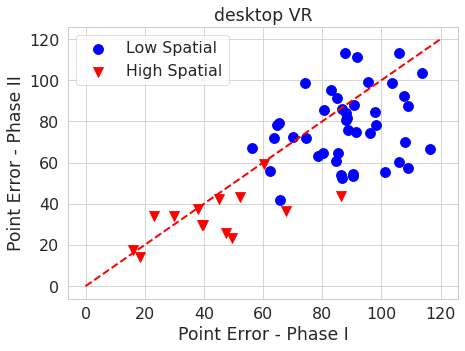


Figure S4: Scatter plots for the Desktop Study Based on the Matched Trials. (a) The relationship between the Pointing Error Phase I and Travel Efficiency (b)The relationship between the Pointing Error Phase I and Phase II

For low-spatial participants, in the desktop study (N=43), the average pointing error before the shortcutting task (88.30 deg, SD = 14.92 deg), was not significantly different from chance (90 deg), one-sample *t* (42) = -0.75, *p* = 0.46, d=-0.11, 95%CI = [83.71, 92.89]. Moreover, these participants’ pointing performance across trials was not reliable (internal consistency = 0.36). However, their average travel efficiency score was 1.98, which was significantly shorter than the learned route (Efficiency = 2.54), one-sample *t*(42) = -13.43, *p*<0.001, *d*= -2.05, 95%CI = [1.90, 2.06], suggesting some ability to take novel efficient paths than the learned route, even though they pointed at chance.

As shown in Table S1, for low spatial participants, the observed correlations between Pointing Error (Phase I) and shortcutting are not significant (*r(41)* = 0.07, *t*(41) = 0.02, *p* = .65, 95%CI = [-.23, .36]). Consistent with the conclusions in the main text, this was partially driven by the low internal consistency of both measures. After correcting for the internal inconsistency of the measured, the disattenuated correlation between the Pointing Error (Phase I) and shortcutting was still not significant (*r_d_(41)* = 0.22, *t*(41) = 1.41, *p* = .17, 95%CI = [-.09, .49]). It suggests that low spatial participants’ pointing performance after the learning phase cannot predict their shortcutting performance.

*Table S4.*

*Descriptive Statistics and Internal Consistency for Measures*

|  | Spatial Ability | Mean | SD | Min | Max | Skew-ness | Kurtosis | # of Trials | internal consistency |
| --- | --- | --- | --- | --- | --- | --- | --- | --- | --- |
| **Desktop** |  |  |  |  |  |  |  |  |  |
| Pointing Error  ( Phase I) | High | 43.79 | 19.36 | 16.16 | 86.21 | 0.50 | -0.12 | 19 | 0.74 |
|  | Low | 88.30 | 14.92 | 56.32 | 116.58 | -0.18 | -0.57 |  | 0.36 |
| Efficiency | High | 1.29 | 0.18 | 1.00 | 1.67 | 0.26 | -0.40 | 19 | 0.57 |
|  | Low | 1.98 | 0.27 | 1.42 | 2.51 | -0.24 | -0.80 |  | 0.29 |
| Pointing  Error  (Phase II) | High | 33.71 | 11.74 | 14.32 | 59.26 | 0.29 | -0.03 | 19 | 0.47 |
|  | Low | 77.06 | 17.80 | 42.05 | 113.11 | 0.25 | -0.65 |  | 0.55 |
